# In Vitro Simulated Ketogenic Diet Inhibits the Proliferation and Migration of Liver Cancer Cells by Reducing Insulin Production and Down-regulating FOXC2 Expression

**DOI:** 10.5152/tjg.2024.23601

**Published:** 2024-09-01

**Authors:** Xiangming Ma, Fei Tian, Jian Li, Zhenyu Wu, Liying Cao

**Affiliations:** 1 Department of Hepatobiliary Surgery, Kailuan General Hospital, Tangshan, Hebei, China; 2 Laboratory of Hepatobiliary, Kailuan General Hospital, Tangshan, Hebei, China; 3 Department of Radiation Oncology, North China University of Science and Technology Affiliated Hospital, Tangshan, Hebei, China; 4 Department of Diagnostic Radiology, Kailuan General Hospital, Tangshan, Hebei, China; 5 Department of Emergency, Kailuan General Hospital, Tangshan, Hebei, China

**Keywords:** Ketogenic diet, insulin, FOXC2, liver cancer

## Abstract

**Background/Aims:**

Ketogenic diet (KD) may benefit patients with liver cancer, but the underlying mechanism of its anti-cancer effect remains an open issue. This work aimed to explore the influence of simulated KD on the proliferation and migration of cultured hepatoma cells.

**Materials and Methods:**

The low-glucose medium supplemented with β-hydroxybutyrate (BHB-G^low^) was utilized to simulate clinical KD treatment. Western blot was utilized for detecting the expression of glycolysis-related proteins, Seahorse XF96 for oxygen consumption rate (OCR) and extracellular acidification rate (ECAR), and ELISA for insulin content. Expression of FOXC2 in liver cancer cells was analyzed by bioinformatics and qPCR. Cell Count Kit-8 (CCK-8) testing kit was utilized for testing cell viability.

**Results:**

KD treatment significantly reduced the expression of glycolysis-related proteins in Huh-7 cells, inhibited insulin production in β islet cells, reduced ECAR, and increased OCR. FOXC2 was significantly up-regulated in Huh-7 cell line, and sh-FOXC2 hindered the proliferation and migration of Huh-7 cells. The exogenous addition of insulin promoted the malignant progression of Huh-7 cells. Together, the medium simulating KD environment strengthened the protection of liver cancer cells by reducing insulin production and down-regulating FOXC2 expression.

**Conclusion:**

This study confirmed through in vitro cell experiments that KD could inhibit the proliferation and migration of liver cancer cells by targeting down regulation of insulin and FOXC2 expression, providing new theoretical basis for the treatment of liver cancer patients.

**Main Points**

- Ketogenic diet was revealed to reduce insulin production by liver cancer cells.
- FOXC2 was highly expressed in liver cancer and promoted the proliferation and migration of liver cancer cells.
- It was confirmed for the first time that the ketogenic diet enhanced the protection of liver cancer patients by reducing insulin and decreasing FOXC2 expression.

## Introduction

Liver cancer ranks as the sixth most prevalent cancer in terms of new cases globally and stands as the third leading cause of mortality, posing a huge threat to people’s health and economic development.^1^ For patients with advanced hepatocellular carcinoma (HCC), there are different treatment strategies, including tyrosine kinase signaling inhibitors, angiogenesis inhibitors, and immune checkpoint inhibitors.^2^ Nevertheless, none of these addresses the modified metabolic function of liver cancer cells. Cancer cells display an atypical metabolic pattern in which they produce lactate from large amounts of pyruvate produced by glycolysis even with sufficient oxygen, which is called the Warburg effect.^3^ Numerous studies of glycolysis-promoting liver cancer progression have been reported. For example, ZEB1 promotes tumorigenesis and metastasis in HCC by facilitating the Warburg effect through transcriptional activation of PFKM.^4^ Knockdown of FOXK1 hinders liver cancer cell activity by suppressing glycolysis.^5^ Hence, targeting glycolytic metabolic pathways could proffer a fresh therapeutic strategy for liver cancer.

Functioning as a high-fat and low-carbohydrate dietary regimen, the ketogenic diet (KD) has been observed to mitigate glucose metabolism while augmenting lipid metabolism, thereby exerting a disruptive influence on the Warburg effect.^1^ Originally utilized to treat intractable epilepsy, KD has recently grown into an underlying metabolic therapy for cancer by converting fatty acids to ketone bodies in a bid to cut down insulin secretion and switch to fat oxidation as fuel.^3^ Accumulating studies prove that KD might be an effective anti-cancer treatment. For example, within a murine model of colon cancer, KD induces anti-tumor properties by triggering oxidative stress, suppressing MMP-9 expression, and rebalancing the M1/M2 tumor-associated macrophage phenotype.^6^ In the BALB/c-nu mouse neuroblastoma xenograft model established by the human neuroblastoma cell line (SH-SY5Y), KD may hinder tumor growth by regulating cell autophagy.^7^ Additionally, some studies have discussed the influence of KD on liver cancer. Wang et al^3^ found that KD up-regulates HMGCS2 expression and hinders the growth of HCC tumor; in the meantime, a negative correlation between tumor size and HMGCS2 expression was observed. Healy et al^8^ pointed out the reduction of KD in tumor burden in mice with HCC. However, Byrne et al^9^ ascertained no significant influence of KD on the progress of HCC in mice. Overall, the mechanism of KD in liver cancer remains an open issue. Hence, it is urgent to investigate the factors influencing the anti-tumor effects in liver cancer, thus providing more insights for precision medicine to develop personalized treatment strategies.

Belonging to the forkhead transcription factor family,^10^ FOXC2 is located on the long arm of chromosome 16, plus strand, consisting of a single exon. It regulates the development of certain systems throughout embryogenesis, especially the lymphatic and vascular systems.^11^ Currently, FOXC2 has been ascertained as an oncogene. As Chen et al^12^ revealed, HCC patients with higher FOXC2 expression have shorter overall survival and FOXC2 stimulates the activation of the Ang-2 promoter. Inhibition of Ang-2 expression impedes FOXC2-mediated EMT, cell migration, and HCC invasion. FOXC2 fosters oxaliplatin resistance in colorectal cancer by triggering epithelial-mesenchymal transition through MAPK/ERK signaling.^13^ Insulin has been ascertained by previous studies to affect the expression of FOXC2. For example, insulin can effectively trigger the expression of FOXC2 protein during the differentiation of adipose tissue-derived mesenchymal stem cells, which may be achieved by regulating the activity of the FOXC2-Pro-512T promoter.^14^ Insulin enhances prostate cancer cells to migrate and invade by up-regulating the expression of FOXC2.^15^ Thus, we speculated that insulin can influence FOXC2 expression in liver cancer cells, which will lead to a promising direction in exploiting the treatment for liver cancer.

In the case of cancer, dysregulation of insulin signaling and overexpression of transcription factor FOXC2 are related to promoting tumor proliferation and migration. Therefore, we hypothesized that simulating a KD in vitro may lead to a decrease in insulin production and downregulation of FOXC2 expression in liver cancer cells, thereby inhibiting liver cancer cell proliferation and migration. This study provided valuable insights for dietary intervention in cancer treatment by studying the mechanism of in vitro simulated KD on cancer cells.

## Materials and Methods

### Bioinformatics Analysis

The mRNA count data of 572 liver cancer patients in The Cancer Genome Atlas (TCGA)-Liver Cancer cohort was acquired by linkage to the National Cancer Center website (https://gdc.cancer.gov/), annotated utilizing GENCODE v22. mRNA expression profile data were normalized by log2 (RSEM+1).^16^

### Cell Culture

Human normal hepatocyte THLE-3 and liver cancer cell line Hep G2 were bought from the American Type Culture Collection (USA). Liver cancer cell lines Huh-7 and Hep3B were bought from the Shanghai Cell Bank of Chinese Academy of Sciences (Shanghai, China), and human β islet cells from Ke Lei Biotechnology Co., Ltd (Shanghai, China). THLE-3 cells were kept in the BEGM medium added with 10% fetal bovine serum (FBS) (HyClone Laboratories, South Logan, UT, USA), 5 ng/mL EGF and 70 ng/mL ethanolamine phosphate. Hep G2 cells and Huh-7 cells were kept in the DMEM medium with 10% FBS. Hep3B cells were cultured in the DMEM medium containing 10% FBS, 1 mL glutamine (Invitrogen, Carlsbad, CA, USA), 1ml nonessential amino acid 100X (Invitrogen), and 1 mL sodium pyruvate 100 mM solution (Invitrogen). Human β islet cells were kept in the RPMI-1640 medium with 10% FBS and 1% streptozotocin. All cells were cultured at 37°C in 5% CO_2_.

To simulate the KD, liver cancer cells Huh-7 were kept in the DMEM medium with 2.5 mM glucose and 10 mM BHB under the same conditions as described above. To identify the influence of insulin on the growth of liver cancer cells, 10 nM insulin was added to the medium during ketogenic treatment. Insulin was purchased from Sigma-Aldrich (St. Louis, MO, USA).

### Cell Transfection

Short hairpin RNA (shRNA) specifically targeting FOXC2 (sh-FOXC2), as well as its corresponding negative control, was procured from GenePharma (Shanghai, China). They were transfected into liver cancer cells by utilizing Lipofectamine 2000 (Invitrogen).

### Quantitative Polymerase Chain Reaction

Total RNA was extracted from cultured cells utilizing TRIzol reagent (Ambion, Austin, TX, USA). Reverse transcription of RNA (2 μg) was done by High-Capacity cDNA Reverse Transcription Kits (Applied Biosystems, Foster City, CA, USA). We added cDNA, KAPA SYBR FAST qPCR Master Mix (2×), and forward/reverse primer mix (Applied Biosystems) to 96-well PCR plates for reaction. The reaction was performed in StepOne system (Applied Biosystems). The cycle threshold (Ct values) was exported to Excel for data analysis. The gene expression level was ascertained by the 2^−△△CT^ method, taking β-actin as the internal reference gene. Table 1 listed the primer sequences.

### Cell Viability and Proliferation

Cell Count Kit-8 (CCK-8) testing kit (Dojindo Molecular Technologies, Tabaru, Japan) was utilized to assay cell viability. Cells were put in 96-well plates (2 × 1042 × 10^4^ cells/well) with the medium containing 2.5 mM glucose and 10 mM BHB. The cells kept in the medium with 25 mM glucose were utilized as controls. The DMEM medium was utilized for sh-NC/sh-FOXC2 treated cells. For analyzing the viability when the culture ended, cells were incubated with 10 µL CCK-8 reaction solution (per 100 µL medium) at 37°C for 40 min at 0 hours, 24 hours, 48 hours, and 72 hours. In the end, the optical density at 450 nm was estimated utilizing a Bio-Rad 680 microplate reader (Bio-Rad Laboratories, Hercules, CA, USA).

5-ethynyl-2’-deoxyuridine (EdU) assay was utilized to assess cell proliferation. In short, cells were kept in the medium with 50 mM EdU (RiboBio, Guangzhou, China) for 2 hours, then at room temperature, fixed with 4% PFA for half an hour, followed by staining with Apollo 567 (RiboBio) for 30 minutes. Positively stained cells were captured utilizing a fluorescence microscope (Olympus, Japan).

### Transwell Migration Assay

Transfected Huh-7 cells were seeded with 4 × 1044 × 10^4^ cells/well, followed by incubation in the serum-free medium. The basolateral chamber was added with the medium containing 10% FBS. After a 24-hour incubation period, non-migratory cells were eliminated. Subsequently, migrated cells were immobilized with 4% methanol for half an hour and subjected to staining using a 0.1% crystal violet for 15 minutes. Images of five random areas were captured by microscope (Carl Zeiss AG, Germany) to number migrated cells.

### Glycolytic Stress Assay

Seahorse XF96 Glycolysis Analyzer (Seahorse Bioscience, North Billerica, MA, USA) was utilized to analyze extracellular acidification rate (ECAR) and oxygen consumption rate (OCR) to assess the effects of KD on glycolytic stress and cellular mitochondrial stress. Glucose, oligomycin, and 2-deoxyglucose were added to the medium in turn for ECAR analysis. First, glucose was injected into the medium, in which it was catabolized to lactate and ATP, with a corresponding rise in ECAR values. Second, oligomycin was introduced, which hindered mitochondrial ATP generation and transferred energy to glycolysis with a rise in ECAR. Extracellular acidification rate was presented in mpH/min units.

Regarding OCR analysis, oligomycin was injected into the medium first. Carbonyl cyano4-(trifluoromethoxy)phenylhydrazone (FCCP) was then introduced, resulting in rapid oxygen consumption. The induced increase in respiratory capacity compared to basal respiration represents spare respiratory capacity. Lastly, rotenone, antimycin A, and electron transport chain inhibitors were injected. Residual respiration corresponded to non-mitochondrial respiration. OCR was presented in pmoL/min units.^17^

### Western Blotting

RIPA lysis buffer (Beyotime Institute of Biotechnology, Shanghai, China) with complete protease inhibitor mixture (Roche Diagnostics, Basel, Switzerland) was utilized to treat cells for half an hour at 4°C to acquire whole cell lysates. The bicinchoninic protein assay kit (Thermo Fisher Scientific, Waltham, MA, USA) was utilized to measure the total protein concentration. Protein samples (20 g) were loaded onto 10% sodium dodecyl sulfate (SDS) gels and separated by SDS-PAGE. Following separation, proteins were transferred to PVDF membranes and blocked with 5% skim milk powder for 4 hours at room temperature. Then, samples were cultured with rabbit anti-human primary antibodies at 4 °C for 12 hours, and after that, cultured with goat anti-rabbit secondary antibody IgG at room temperature for 2 hours. Signals were observed utilizing an enhanced chemiluminescence detection system (GE Healthcare, Little Chalfont, Buckinghamshire, UK). Primary antibodies utilized included: β-actin (ab8226, 1 : 1000, Abcam, Cambridgeshire, East of England, UK), HK2 (ab209847, 1 : 1000, Abcam), PKM2 (ab85555, 1 : 1000, Abcam), and LDHA (ab52488, 1 : 5000, Abcam).

### Insulin Measurement

For insulin assay of cell supernatant, the cell supernatant was taken directly for culture, measured by using the Human Insulin ELISA assay kit (Crystal Chem, Cook, Illinois, USA) after centrifugation.^18^

### Statistical Analysis

Data are expressed as mean ± SD. GraphPad Prism version (GraphPad Software, USA) was utilized for all data analyses. Statistical analysis was fulfilled by utilizing repeated-measures analysis of variance and post hoc Tukey test. The difference between the two groups was analyzed by a two-sample *t*-test. *P *< .05 indicated statistical significance.

## Results

### BHB-G^low^ Medium Hinders Glycolysis and FOXC2 Expression in Liver Cancer Cells

**Figure 1.**
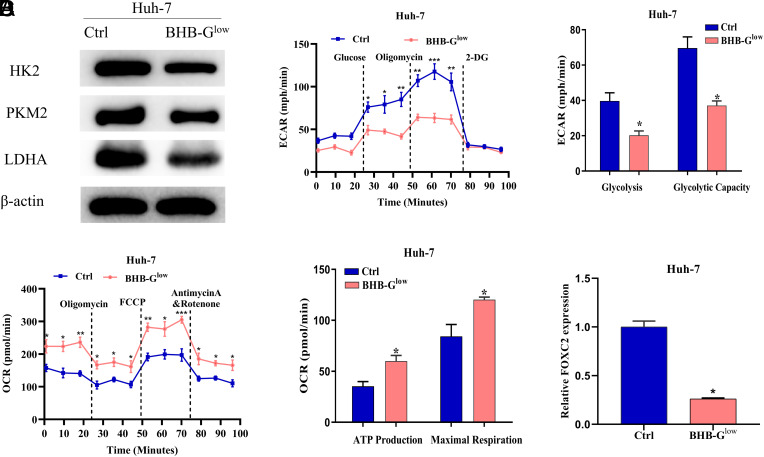
BHB-G^low^ medium hinders glycolysis and FOXC2 expression in liver cancer cells. (A) Expression levels of glycolysis-related proteins after ketogenic treatment; (B and C) Seahorse XF96 glycolysis analyzer was utilized to analyze ECAR and OCR of different groups of cells; (D) FOXC2 expression after ketogenic treatment by qPCR, *P < .05; **P< .01; ***P< .001. ECAR, extracellular acidification rate; OCR, oxygen consumption rate.

To investigate the impact of KD on the metabolic pattern of liver cancer cells, we cultured Huh-7 cells using BHB-G^low^ medium (2.5 mM glucose with the addition of 10 mM BHB) as a way to simulate clinical ketogenic treatment, and the control was normal medium containing 25 mM glucose. WB detected the expression of glycolysis-related proteins HK2, PKM2, and LDHA, with results showing that BHB-G^low^ medium tellingly decreased the expression of the above proteins (Figure 1A). Subsequently, we found that ketogenic treatment significantly decreased the ECAR value and increased OCR value of cells (Figure 1B and1C), indicating that ketogenic treatment was able to inhibit the glycolytic process in liver cancer cells. Furthermore, KD was found to affect insulin levels,^1^ while insulin enhances cancer cells to migrate and invade by up-regulating FOXC2,^15^ we hypothesized that BHB-G^low^ medium might affect FOXC2 expression. To test this hypothesis, we examined FOXC2 expression after ketogenic treatment and found that FOXC2 expression was significantly hindered (Figure 1D). Based on the above results, a simulated KD environment could regulate liver cancer cell metabolism, and affect the expression of FOXC2.

### FOXC2 has High Expression in Liver Cancer and Fosters the Proliferation and Migration of Liver Cancer Cells

**Figure 2.**
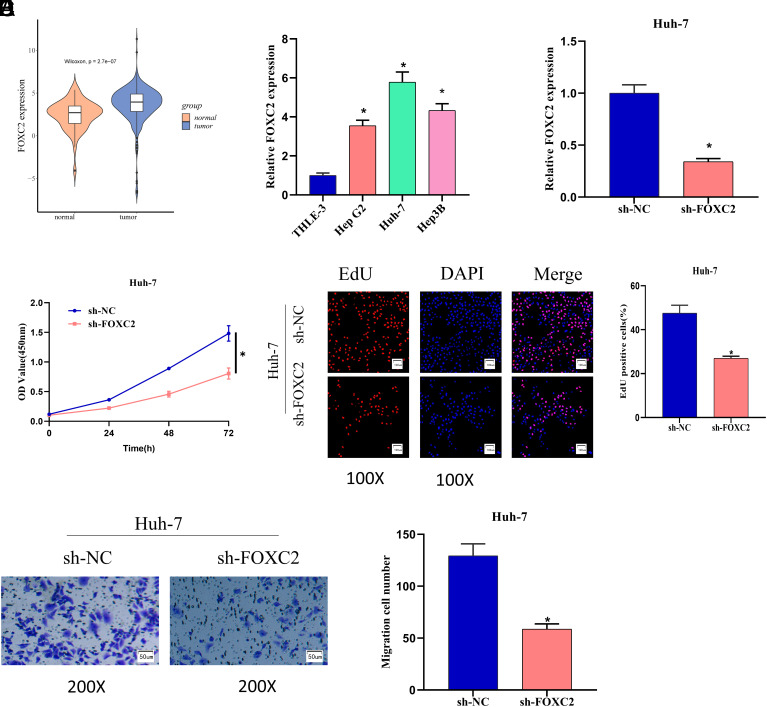
FOXC2 expression is up-regulated in liver cancer and promotes cell proliferation and migration. (A) Bioinformatics analysis of FOXC2 expression in liver cancer tissues (orange: normal tissues; blue: cancer tissue); (B) quantitative polymerase chain reaction (qPCR) was utilized to detect the expression of FOXC2 in liver cancer cells Hep G2, Huh-7, Hep3B, and normal cells THLE-3; (C) qPCR was utilized to detect the expression of FOXC2 after sh-FOXC2 treatment; (D) CCK-8 was utilized to detect the viability of Huh-7 cells after sh-FOXC2 treatment; (E and F) EdU (100×) and Transwell (200×) assays were utilized to detect the proliferation and migration ability of Huh-7 cells after sh-FOXC2 treatment, *P < .05.

The above studies ascertained that the BHB-G^low^ medium affected the expression of FOXC2. Therefore, FOXC2 was selected as the research object here. We found significant up-regulation of FOXC2 in liver cancer tissues by bioinformatics analysis, and high expression of FOXC2 in liver cancer cells by qPCR (Figure 2A and 2B). Since FOXC2 was relatively highly expressed in Huh-7 cells, we chose this cell line for subsequent experiments. We then constructed the following groups based on the Huh-7 cell line: sh-NC and sh-FOXC2. As qPCR and CCK-8 results showed, sh-FOXC2 treatment significantly reduced FOXC2 expression and cell viability (Figure 2C and 2D). EdU and Transwell assays were utilized to assay the proliferation and migration of cells after different treatments, with results showing that the proliferation and migration ability of Huh-7 cells in the sh-FOXC2 group was significantly reduced (Figure 2E and 2F). Together, FOXC2 was considerably up-regulated in liver cancer and could foster liver cancer cells to migrate and proliferate.

### Increased Insulin Promotes the Proliferation and Migration of Liver Cancer Cells

**Figure 3.**
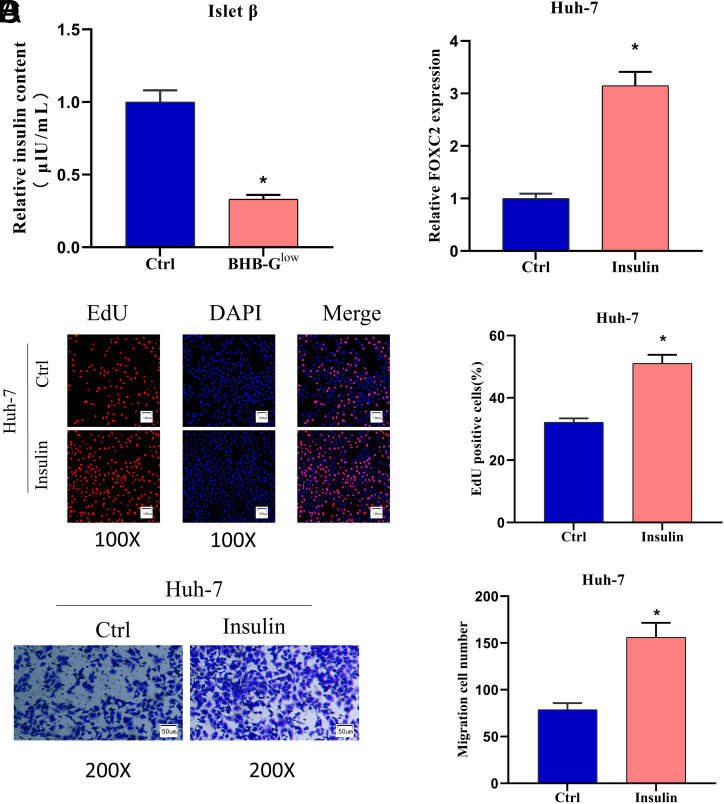
Increased insulin promotes the proliferation and migration of liver cancer cells. (A) ELISA was used to detect the effect of ketogenic treatment on insulin release from β islet cells; (B) qPCR was used to detect the effect of insulin on FOXC2 expression; (C) EdU was used to detect the effect of insulin treatment on the proliferation of liver cancer cells (100×); (D) Transwell assay was used to detect the effect of insulin treatment on the migration ability of liver cancer cells (200×); *P < .05.

To figure out the underlying mechanism of KD affecting liver cancer cell growth, we measured insulin secretion by ELISA in pancreatic β cells cultured in the BHB-G^low^ medium KD and found that insulin content was significantly decreased in the ketogenic group versus control group (Figure 3A). To figure out the influence of insulin secretion on liver cancer cells, we treated liver cancer cells by adding 10 nM insulin to the medium. FOXC2 expression was detected by qPCR under different culture conditions and was found to be enhanced by the addition of insulin (Figure 3B). We then investigated how insulin affected the malignant behavior of liver cancer cells and found that insulin promoted the proliferation and migration of Huh-7 cells (Figure 3C and 3D). The BHB-G^low^ medium was ascertained by these outcomes to foster the malignant development of liver cancer cells through insulin release from β islet cells.

### BHB-G^low^ Medium Can Hinder the Proliferation and Migration of Liver Cancer Cells by Suppressing the Production of Insulin

**Figure 4.**
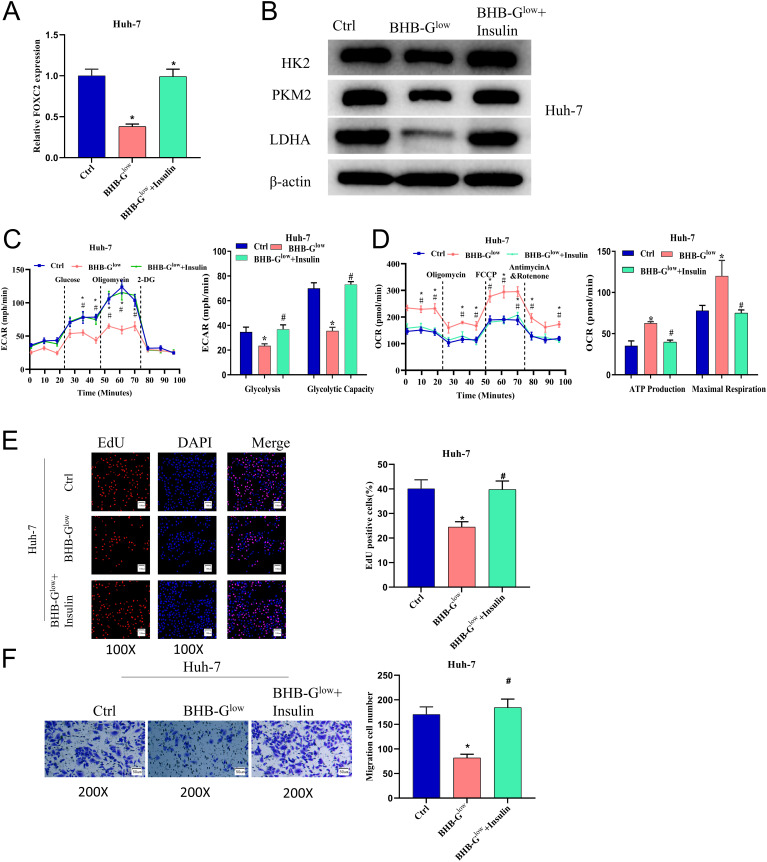
BHB-G^low^ medium can hinder the proliferation and migration of liver cancer cells by suppressing the production of insulin. (A) qPCR was used to detect the expression of FOXC2 in Huh-7 cells of different groups; (B) WB was used to detect the expression of HK2, PKM2, and LDHA in Huh-7 cells of different groups; (C and D) Seahorse XF96 glycolysis analyzer was used to analyze ECAR and OCR of Huh-7 cells in different groups; (E and F) EdU (100×) and Transwell (200×) assays were used to detect the proliferation and migration ability of Huh-7 cells after different treatments (*VS Ctrl, #VS BHB-G^low^, *P *< .05).

To investigate how BHB-G^low^ medium affected liver cancer cells, we added 10 nM insulin to Huh-7 cells under ketogenic treatment. Expression of FOXC2 under different culture conditions was detected by qPCR, with results showing that ketogenic treatment could hinder the expression of FOXC2, and the addition of insulin restored FOXC2 to the control level (Figure 4A). WB was utilized to assay the expression of HK2, PKM2, and LDHA in each group, and it was found that insulin reversed the down-regulated expression of the above proteins caused by ketogenic treatment (Figure 4B). Seahorse XF96 glycolysis analyzer examined the ECAR values and came out the same result (Figure 4C), whereas the OCR results were opposite (Figure 4D). Next, we investigated the influence of ketogenic treatment on the malignant behavior of liver cancer cells, and the proliferation and migration abilities of Huh-7 cells were found weakened after ketogenic treatment, which was reversed by the addition of insulin (Figure 4E and 4F). In conclusion, the BHB-G^low^ medium was able to hinder the expression of FOXC2 by hindering insulin production, thereby suppressing the proliferation and migration of liver cancer cells.

## Discussion

Liver cancer remains one of the most prevalent and fatal cancers worldwide.^19^ With limited treatment options for liver cancer in the past decades, there is an imperative need to develop new treatments to improve the survival status of clinical liver cancer patients. In recent years, KD has attracted researchers’ attention as a way to target catabolic differences between normal and cancer cells.^3^ Ketogenic diet is a therapy that alters the composition of the diet and is safer than medication.^1^ Generally, most clinical and preclinical studies have illustrated beneficial anticancer effects of KD, such as a randomized study using ketogenic metabolic therapy for breast cancer patients, which found lower serum insulin and reduced tumor size (27 vs. 6 mm) in the ketogenic group, with a meaningful decrease in staging after 12 weeks in patients with locally advanced disease.^20^ In the colon cancer mouse model, mice in the KD group had longer survival time and better health, significantly smaller weight of ascites, and improved anemia symptoms, red blood cell count, hemoglobin count as well as hematocrit.^21^ However, KD has been shown to have no effect on reducing tumor volume in several studies on gliomas and medulloblastomas.^22,23^ Apparently, tissue specificity might determine whether a tumor is sensitive to KD therapy. Hence, this work investigated how KD affected liver cancer progression to pave the way for relevant treatment through in vitro experiments.

In this study, the BHB-G^low^ medium simulating KD environment in vitro was found to inhibit insulin production in liver cancer cells. Insulin is a protein hormone secreted by the pancreatic β cells in the pancreas in response to incentive by a variety of internal and external substances. It is the only glucose-lowering hormone in the body, so it promotes the synthesis of protein, fat, and glycogen.^24^ Recent studies have revealed that insulin features in the survival and growth of cancer cells.^25^ Cao et al^26^ ascertained the extracellular vesicles secreted by breast cancer cells hinder insulin secretion via miR-122, thereby impairing systemic glucose homeostasis and promoting tumor growth. Coincidentally, biopsies from colon cancer patients have shown that hyperglycemia is associated with ACAT1, lymph node metastasis, and distant metastasis. Insulin significantly promotes human colon cancer HT-29 cells to proliferate and migrate.^27^ Furthermore, accumulating evidence shows that the influence of insulin on tumor growth is carried out by changing the expression of oncogenes or tumor suppressor genes, like insulin-induced programmed death ligand 1 (PD-L1) expression in pancreatic ductal adenocarcinoma (PDAC) cells, thus suppressing the activity of CD8+T cells in PDAC.^27^ As Heckl et al^28^ revealed, insulin promotes the progression of colon cancer by up-regulating the expression of ACAT1. Our results produced similar findings compared to previous studies. In liver cancer, KD hindered the expression of FOXC2 by reducing insulin production, thereby repressing the proliferation and migration of liver cancer cells in vitro.

In summary, our work proposed a feasible therapeutic modality in vitro that could be rapidly transformed for targeting liver cancer. Specifically, it was a metabolic regulation at the systemic level, in which the BHB-G^low^ medium hindered FOXC2 expression by interfering with glycolytic metabolism and inhibiting insulin production, thereby inhibiting liver cancer cells’ malignant progression. This implied KD to be a treatment modality for liver cancer. However, our research has limitations as we only conducted in vitro experiments and did not validate the accuracy of the results at the clinical level. In the future, we will collect clinical samples for an in-depth study on KD molecular mechanisms for treating liver cancer. All in all, our work proffered a therapeutic approach when treating liver cancer, and KD, as a safe and effective treatment, may hold significant promise in ameliorating the outcome of patients with liver cancer if combined with existing treatments.

## Figures and Tables

**Table 1.** Quantitative Polymerase Chain Reaction Primer Sequences

Primer	Forward	Reverse
FOXC2	5’-CACAGCGGGGACCTGAA-3’	5’-CAGCCGGTGGGAGTTGA-3’
β-Actin	5’-TGTAGTCCGCACCACCGTAGC-3’	5’-TGTAGTCCGCACCACCGTAGC-3’
